# Association of *DTNBP1* With Schizophrenia: Findings From Two Independent Samples of Han Chinese Population

**DOI:** 10.3389/fpsyt.2020.00446

**Published:** 2020-05-25

**Authors:** Yongfeng Yang, Luwen Zhang, Dong Guo, Lin Zhang, Hongyan Yu, Qing Liu, Xi Su, Minglong Shao, Men Song, Yan Zhang, Minli Ding, Yanli Lu, Bing Liu, Wenqiang Li, Weihua Yue, Xiaoduo Fan, Ge Yang, Luxian Lv

**Affiliations:** ^1^The Second Affiliated Hospital of Xinxiang Medical University, Henan Mental Hospital, Xinxiang, China; ^2^Henan Key Lab of Biological Psychiatry, Xinxiang Medical University, Xinxiang, China; ^3^Psychiatry Department, Affiliated Wuhan Mental Health Center, Tongji Medical College of Huazhong University of Science & Technology, Wuhan, China; ^4^Affiliated Wuhan Mental Health Center, Tongji Medical College of Huazhong University of Science & Technology, Wuhan, China; ^5^Brainnetome Center, Institute of Automation, Chinese Academy of Sciences, Beijing, China; ^6^National Laboratory of Pattern Recognition, Institute of Automation, Chinese Academy of Sciences, Beijing, China; ^7^University of Chinese Academy of Sciences, Beijing, China; ^8^Institute of Mental Health, Peking University, Beijing, China; ^9^Ministry of Health Key Laboratory of Mental Health, Peking University, Beijing, China; ^10^Psychiatry Department, University of Massachusetts Medical School and UMass Memorial Medical Center, Worcester, MA, United States; ^11^Psychiatry Department, Henan Provincial People's Hospital, Zhengzhou, China

**Keywords:** schizophrenia, *DTNBP1*, polymorphism, psychotic symptoms, cognition

## Abstract

**Objectives:**

Schizophrenia (SZ) is a complex psychiatric disorder that has a strong genetic basis. Dystrobrevin-binding protein 1 (*DTNBP1*) is one of the genes thought to be pivotal in regulating the glutamatergic system. Studies have suggested that variations in *DTNBP1* confer susceptibility to SZ and clinical symptoms. Here, we performed a two-stage independent verification study to identify polymorphisms of the *DTNBP1* gene that might be associated with SZ in the Han Chinese population.

**Methods:**

In stage 1, 14 single nucleotide polymorphisms (SNPs) were genotyped in 528 paranoid SZ patients and 528 healthy controls (HCs) using the Illumina GoldenGate assays on a BeadStation 500G Genotyping System. In stage 2, ten SNPs were genotyped in an independent sample of 1,031 SZ patients and 621 HCs using the Illumina 660k Genotyping System. Clinical symptoms were assessed using the Positive and Negative Syndrome Scale.

**Results:**

There was a significant association related to allele frequency, and a trend association in relation to genotype between SZ patients and HCs at rs4712253 (*p* = 0.03 and 0.05, respectively). These associations were not evident following Bonferroni correction (*p >* 0.05 for both). Haplotype association analysis revealed that only two haplotypes (GAG and GAA; rs16876575-rs9464793-rs4712253) were significantly different between SZ patients and HCs (*χ^2^* = 4.24, 6.37, *p* = 0.04 and 0.01, respectively). In addition, in SZ patients there was a significant association in the rs4964793 genotype for positive symptoms, and in the rs1011313 genotype for excitement/hostility symptoms (*p* = 0.01 and 0.002, respectively). We found a significant association in the baseline symbol digital modalities test (SDMT), forward-digital span (DS), backward-DS, and semantic fluency between SZ patients and HCs (*p <* 0.05 for all). Finally, the SNP rs1011313 genotypes were associated with SDMT in SZ patients (*p* = 0.04).

**Conclusion:**

This study provides further evidence that SNP rs4712253 of *DTNBP1* has a nominal association with SZ in the Han Chinese population. Such a genotype variation may play a role in psychopathology and cognitive function.

## Introduction

Schizophrenia (SZ) is a chronic and complex mental illness, with up to 1% of the world's population being affected (1). Although the etiology of SZ has not been fully elucidated, longstanding evidence has shown that SZ is mainly a genetic disorder, with up to 80% heritability (2, 3). SZ genetic research often focuses on identifying linkage regions, candidate genes and single nucleotide polymorphisms (SNPs). A genome wide association study with a large sample size found 108 susceptibility loci associated with SZ, underscoring the complex genetic component of SZ (4). Some previous studies have suggested that multiple individual mutations could alter genes in neurotransmitter pathways such as the glutamatergic system, which contribute to the development of SZ (5, 6).

As one of the primary excitatory neurotransmitters, glutamate plays an important role in neuronal development, synaptic plasticity, and neurotoxicity in the central nervous system (7). Glutamatergic neurotransmission dysfunction might be associated with negative symptoms and cognitive symptoms of SZ (8, 9). The glutamatergic pathway has been considered as a new therapeutic target, in addition to the dopaminergic pathway, for SZ (10). The gene encoding dystrobrevin-binding protein 1 (*DTNBP1*, locus: 6p24-21) was reported as a susceptibility factor for SZ (11) and was initially identified through multipoint linkage analysis of Irish high-density pedigrees (12, 13). Based on results of previous studies, some researchers believed that the neural pathway involved in *DTNBP1* may constitute a potential therapeutic target for the treatment of SZ (10, 14), and suggested that *DTNBP1* plays a role underlying the etiology of SZ.

Further studies have supported the role of *DTNBP1* as a susceptibility gene of SZ. Li et al. found a correlation between SNP rs2619528 and SZ in 638 nuclear families in the Chinese Han families (15). Additional studies reported an association between SNP rs3213207 and SZ in Norwegian (155 patients), Spanish (589 patients vs 615 controls), and Chinese (638 nuclear families) populations (8, 15, 16). SNPs rs9370822, rs1997679, and rs4236176 were found to be associated with SZ in the Caucasian population (160 patients vs 259 controls) (17). In addition, a meta-analysis confirmed a strong correlation between *DTNBP1* and SZ (18). Previous studies found a protective effect of the rs6459409 SNP and the estimated/phased CT diplotype (rs6459409-rs9476886) in males for the development of SZ (14), as well as a significant association between rs2005976 and rs2691528, and between rs2005976 and rs760761 in Chinese (638 nuclear families) and Scottish (580 patients vs 620 controls) populations, respectively (15). However, some inconsistent results have been reported. For example, rs3213207 was found not to be a susceptibility site in UK and Korean populations (19, 20). Some studies reported that the SNPs rs742106, rs3829893, rs4712253, rs9476886, rs1011313, and rs2619539 were not susceptibility sites in different populations (15–17, 19–21); these findings were confirmed in a meta-analysis (18).

To further clarify the role of DTNBP1 in SZ, we carried out a case-control study to investigate potential associations among *DTNBP1* SNPs, and to explore the correlation between genotype and psychopathology and cognition in the Han Chinese population.

## Materials and Methods

### Subjects

Regulations governing this research were based on the principles of the World Medical Association Declaration of Helsinki and the Ethics Committee of the Second Affiliated Hospital of Xinxiang Medical University (Xinxiang, China). All participants involved in this study signed an informed consent form.

Participants were recruited from the Second Affiliated Hospital of Xinxiang Medical University. We included a total of 1,559 patients with SZ (stage 1 *vs* 2 = 528 vs 1,031) and 1,149 healthy controls (HCs, stage 1 *vs* 2 = 528 vs 621). All patients were born and lived in the northern part of Henan Province, China. All of their biological grandparents and parents were of Chinese Han origin. HCs were from the same geographic areas; the inclusion and exclusion criteria of HCs were the same as reported in our previous studies (5, 22).

The diagnostic criteria for SZ were based on the Diagnostic and Statistical Manual of Mental Disorders-Fourth Edition. The psychopathology of SZ was assessed using the Positive and Negative Syndrome Scale (PANSS) (23) that includes five factors (24). Evaluation of antipsychotic efficacy after 6 weeks of treatment of SZ patients were assessed to determine the reduction in the PANSS score; the responder group exhibited a >50% reduction and the non-responder group exhibited a ≤50% reduction (25). Cognitive function in SZ patients was assessed using the symbol digital modalities test (SDMT), and also with forward-digital span (DS), backward-DS, and semantic fluency.

### Genotyping

Peripheral blood samples were collected from all subjects using evacuated tubes containing EDTA anticoagulant. Genomic DNA was extracted from white blood cells using the RelaxGene Blood DNA System (Tiangen Biotech, Beijing, China). Genotyping was performed using Illumina GoldenGate assays on a BeadStation 500G Genotyping System (Illumina, San Diego, CA, USA) in stage 1 per our previous studies (5, 22, 26). The llumina 660K Genotyping System was using to genotyping in stage 2.

### Statistical Analyses

Statistical analyses were described in detail elsewhere (5, 22, 26). The Hardy–Weinberg equilibrium (HWE), one of most important principles of population genetics, was used to evaluate the genetic composition and differences in the study populations. Specifically, inbreeding, population stratification, and selection can be induced by the deviations of the HWE (27, 28). Minor allele frequency (MAF) is the frequency of the second most common allele in a given population, and the MAF thresholds strongly affect population structure (29). G*Power software was used to calculate power (http://www.gpower.hhu.de/). The online software SNPStats (30) was used to analysis sex/genotype interactions (https://www.snpstats.net/preproc.php). Haploview V4.1 was used to assess genotypes, and allele and haplotype frequency (31). Associations analyses between different genotypes and five factors of PANSS were performed using one-way analysis of variance (ANOVA) with age, age at illness onset and illness duration as covariables (SPSS version 25.0, IBM Inc., Armonk, NY, USA). *P <* 0.05 was considered statistically significant. Bonferroni corrections were used for multiple comparisons.

## Results

### Demographic Characteristics and Clinical Information

To identify allelic variants of the *DTNBP1* gene that were associated with SZ in the Chinese Han population, allele and genotype frequencies of 14 SNPs in stage 1, and ten SNPs in stage 2, were analyzed. As shown in **Table 1**, a total of 1,559 SZ patients (stage 1 vs 2 = 528:1,031) and 1149 (stage 1 vs 2 = 528:621) HCs were recruited in this study. There were no significant differences in the two groups in respect to either age and gender in stage 1 (*p* = 0.95 and 1.00, respectively) or in respect to gender in stages 1 and 2 combined (*p* = 0.21). However, there was a significant difference between the two groups in respect to age for combined samples (*p <* 0.01). Significant differences between the two groups were also found in respect to family history in stage 1 and in the combined samples (*p <*0.01 for both). For a power above 80%, the effect size is 0.2, and 310 SZ patients and 310 HCs are needed. In stage 1, power analyses revealed that the total sample size (n = 1,056, SZ patients vs HCs = 528:528) had a power of 0.94 to detect a small effect (r = 0.2) for genotype frequency. The sample size (n = 2,112) had a power of 0.99 to detect a small effect (r = 0.2) for allele frequency. Therefore, there was also strong statistical power in stage 2 (n =1,652, SZ vs HCs = 1,031:621).

**Table 1:** Demographic characteristics of the schizophrenia and healthy controls.

Variables	S1			S1&S2		
	SZ	HCs	*P* value	SZ	HCs	*P* value
N	528	528		1559	1149	
Age (years)	27.32 ± 8.03	27.73 ± 8.01	0.95	21.27 ± 14.57	23.56 ± 18.81	<0.01
Age of Oneset (years)	23.47 ± 8.26	NA		16.09 ± 12.06	NA	
Duration of Illness (years)	6.18 ± 5.91	NA		7.69 ± 7.46	NA	
Gender (male/female)			1.00			0.21
Male	264	264		801	552	
Female	264	264		758	597	
Family history			<0.01			<0.01
Yes	82	0		264	0	
No	446	528		1,295	1,149	
Diagnosis Subtype						
Paranoid	528	NA		1,335	NA	
Undifferentiated	0	NA		197	NA	
Catatonic	0			15		
Residual	0			12		

S1, Stage 1; S2, Stage 2; SZ, Schizophrenia, HC, health controls.

### Genotype Analysis

The genotype distribution of the SNPs did not significantly deviate from the HWE except for SNP rs1011313 in stage 1 SZ patients, and rs9464793 in stage 2 SZ patients. In stage 1, we found a statistically significant association in respect to allele frequency and genotype for SNP rs742106 between SZ patients and HCs (*p* = 0.02 and 0.008, respectively; **Table 2**). The associations disappeared after Bonferroni's correction (*p >* 0.05 for both). After breaking down the cohort by gender, the above associations were only found in males (*χ*^2^ = 12.11, *p* = 0.002). We also found significant gender/genotype interaction at SNP rs742106 (*p* = 0.029). However, there were no significant associations in respect to allele frequency and genotype at the other 13 SNPs (rs16876575, rs9464793, rs4712253, rs9370823, rs9358063, rs3829893, rs1011313, rs2619533, rs4715986, rs12199640, rs2619539, rs2619542, and rs9476886) between SZ patients and HCs (*p >* 0.1 for all).

**Table 2:** Genotype and allele frequencies of 14 SNPs in the *DTNBP1* gene in SZ patients and HCs.

SNP#	dbSNP ID	Allele (D/d)^a^	SZ						HCs						*p*-value	
			n^b^	HWE(p)	Genotype			MAF	n^b^	HWE(p)	Genotype			MAF		
					DD	Dd	dd				DD	Dd	dd		Genotype	Allele
1	rs742106	A/G	528	0.73	153	266	109	0.46	528	0.60	192	248	88	0.40	0.02(0.56)*	0.01(0.22)*
2	rs16876575	G/A	527	0.53	373	143	11	0.16	528	0.35	362	154	12	0.17	0.73	0.45
3	rs9464793	A/G	527	0.52	449	76	2	0.08	528	0.09	444	77	7	0.09	0.24	0.39
4	rs4712253	G/A	527	0.34	175	267	86	0.42	528	0.87	201	251	76	0.38	0.34	0.11
5	rs9370823	G/A	528	0.22	130	278	120	0.49	528	0.52	149	256	123	0.47	0.32	0.48
6	rs9358063	G/A	527	0.14	405	118	4	0.12	525	0.29	395	120	13	0.14	0.09	0.20
7	rs3829893	G/A	528	0.92	325	179	24	0.21	527	0.81	330	176	22	0.21	0.93	0.71
8	rs1011313	G/A	527	0.00	306	185	36	0.24	528	0.80	320	187	21	0.22	0.15	0.12
9	rs2619533	A/T	526	0.03	449	70	7	0.08	528	0.09	444	77	7	0.09	0.83	0.60
10	rs4715986	T/A	524	0.57	255	225	44	0.30	527	0.73	260	218	49	0.30	0.81	0.95
11	rs12199640	G/A	528	0.36	357	158	13	0.17	528	0.63	374	139	15	0.16	0.41	0.38
12	rs2619539	C/G	527	0.43	256	228	43	0.30	528	0.83	261	219	48	0.30	0.78	0.98
13	rs2619542	A/G	528	0.30	255	231	42	0.30	527	0.84	260	219	48	0.30	0.68	0.98
14	rs9476886	G/A	528	0.13	238	244	46	0.32	528	0.84	233	237	58	0.33	0.46	0.43

SZ, Schizophrenia; HCs, health controls.

a Major/minor allele, major and minor alleles are denoted by D and d, respectively.

b Number of samples with well genotype.

*p value of Bonferroni correction.

We were not able to find significant associations between SZ and HCs at SNP rs742106 in stage 2 (*p* = 0.72 and 0.61, respectively; **Table 3**). In addition, there were no significant associations in respect to allele frequency and genotype at the other nine SNPs (rs16876575, rs9464793, rs4712253, rs9370823, rs9358063, rs3829893, rs1011313, rs1047631, and rs1997679) between SZ and HCs in the stage 2 (*p >* 0.1 for all). Furthermore, no significant association for gender between SZ and HCs in stage 2 was found (*p >* 0.05).

**Table 3:** Genotype and allele frequencies of 10 SNPs in the *DTNBP1* gene in SZ patients and HCs.

SNP#	dbSNP ID	Allele (D/d)^a^	Stage	SZ						HCs						*p*-value	
				n^b^	HWE(*p*)	Genotype			MAF	n^b^	HWE(*p*)	Genotype			MAF		
						DD	Dd	dd				DD	Dd	dd		Genotype	Allele
1	**rs742106**	A/G	1	528	0.73	153	266	109	0.46	528	0.60	192	248	88	0.40	0.02 (0.56)*	0.01(0.22)*
			2	1,061	0.69	335	528	198	0.43	621	0.28	185	320	116	0.44	0.72	0.61
			1&2	1,589	0.62	488	794	307	0.44	1,149	0.69	377	568	204	0.42	0.40	0.18
2	rs16876575	G/A	1	527	0.53	373	143	11	0.16	528	0.35	362	154	12	0.17	0.73	0.45
			2	340	0.98	238	93	9	0.16	621	0.28	449	162	10	0.15	0.47	0.33
			1&2	867	0.62	611	236	20	0.16	1,149	0.17	811	316	22	0.16	0.83	0.83
3	rs9464793	A/G	1	527	0.52	449	76	2	0.08	528	0.09	444	77	7	0.09	0.24	0.39
			2	341	0.00	192	106	43	0.28	621	0.00	388	158	75	0.25	0.14	0.11
			1&2	868	0.00	641	182	45	0.16	1,149	0.00	832	235	82	0.17	0.62	0.15
4	rs4712253	G/A	1	527	0.34	175	267	86	0.42	528	0.87	201	251	76	0.38	0.34	0.11
			2	1,061	0.20	348	537	176	0.42	621	0.79	228	294	99	0.40	0.26	0.19
			1&2	1,588	0.11	523	804	262	0.42	1,149	0.93	429	545	175	0.39	0.05 (0.80)*	0.03 (0.48)*
5	rs9370823	G/A	1	528	0.22	130	278	120	0.49	528	0.52	149	256	123	0.47	0.32	0.48
			2	1,062	0.81	264	527	271	0.50	618	0.89	168	310	140	0.48	0.34	0.15
			1&2	1,590	0.62	394	805	391	0.50	1,146	0.73	317	566	263	0.47	0.21	0.10
6	rs9358063	G/A	1	527	0.14	405	118	4	0.12	525	0.29	395	120	13	0.14	0.09	0.20
			2	339	0.21	255	81	3	0.13	618	0.59	482	126	10	0.12	0.31	0.51
			1&2	866	0.06	660	199	7	0.12	1,143	0.24	877	246	23	0.13	0.07	0.67
7	rs3829893	G/A	1	528	0.92	325	179	24	0.21	527	0.81	330	176	22	0.21	0.93	0.71
			2	1,061	0.84	648	363	49	0.22	621	0.60	403	197	21	0.19	0.30	0.08
			1&2	1,589	0.82	973	542	73	0.22	1,148	0.60	733	373	43	0.20	0.37	0.13
8	rs1011313	G/A	1	527	0.00	306	185	36	0.24	528	0.80	320	187	21	0.22	0.15	0.12
			2	1,060	0.68	613	383	64	0.24	621	0.44	366	217	38	0.24	0.88	0.74
			1&2	1,587	0.33	919	568	100	0.24	1,149	0.96	686	404	59	0.23	0.37	0.20
9	rs1047631	A/G	2	1,062	0.52	1,021	41	0	0.02	621	0.64	598	23	0	0.02	0.87	0.87
10	rs1997679	G/A	2	1,061	0.58	890	165	6	0.08	620	0.92	516	99	5	0.09	0.81	0.65

SZ, Schizophrenia; HCs, Health controls; MAF, Minor allele frequency; HWE, Hardy–Weinberg equilibrium; SNP, Single nucleotide polymorphism.

a Major/minor allele, major and minor alleles are denoted by D and d, respectively.

b Number of samples with well genotype.

*p value of Bonferroni correction.

After combining stages 1 and 2, there were no significant associations for allele and genotype at SNP rs742106 (*p* = 0.40 and 0.18; respectively). However, there was significant association in respect to allele frequency at SNP rs4712253 between SZ patients and HCs in stages 1 and 2 combined (*p* = 0.03). Meanwhile, there was a trend association in respect to genotype at SNP rs4712253 between two groups in stages 1 and 2 combined (*p* = 0.05). The association disappeared after employing Bonferroni's correction (*p >* 0.05). After breaking down the cohort by gender, there was a significant association in respect to genotype at SNPs rs4712253 and rs9370823 for males, and SNP rs2619539 for females between SZ patients and HCs in stages 1 and 2 combined (*χ*^2^ = 11.10, 8.10, and 8.49; *p* =0.004, 0.017, and 0.014, respectively; **data not shown**). We also found significant gender/genotype interaction at SNP rs4712253, rs9370823, and rs2619539 in stages 1 and 2 combined (*p* = 0.024, 0.048, and 0.041, respectively). After breaking down the cohort by family history of SZ, there was a significant difference at SNP rs9464793 in stages 1 and 2 combined (*χ^2^* = 8.04, *p* = 0.018, **data not shown**).

### Haplotype Analysis

**Figure 1:**
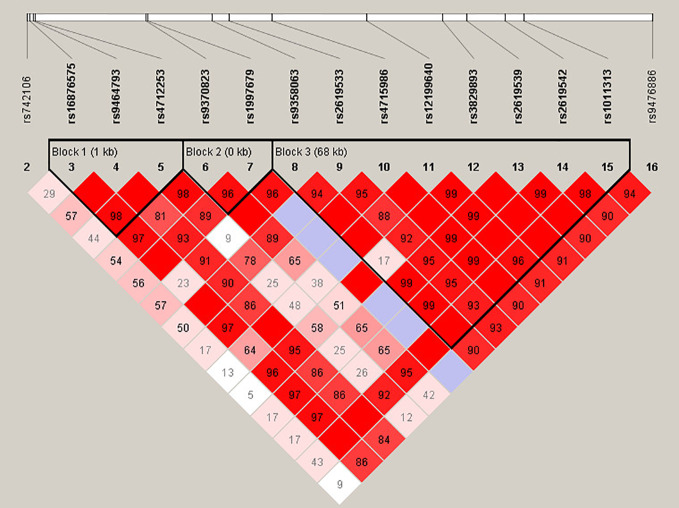
Haplotype block structure of the DTNBP1 gene in both SZ patients and HCs in stage 1 and 2 samplese combined. The index association SNP is represented by a diamond. The colors of the remaining SNPs (circles) indicate LD with the index SNP based on pairwise r2 values from our data.

For more in-depth analysis of the haplotype structure of stages 1 and 2 combined, standardized D′ and *r*^2^ values were used to evaluate the pairwise LD of 16 SNPs in SZ patients and HCs. Haplotypes were formed by 16 SNPs of *DTNBP1*. The locations of these SNPs in *DTNBP1*, the LD structure, and the D′ values for all variants are shown in **Figure 1**. Sixteen SNPs formed three LD blocks and 12 haplotypes; only haplotype GAG and GAA (rs16876575-rs9464793-rs4712253) significantly differed between SZ patients and HCs (*p* = 0.04 and 0.01, respectively, **Supplementary Table 1**).

### Genotype Variation and Psychopathology Analysis

To explore the associations between SNP genotype variations and psychopathology, we selected 672 SZ patients (sample 1 *vs* 2 = 228 vs 444) who had PANSS scores. Among the five PANSS factors, we found that positive symptom scores were significantly different at rs9464793 between the GG and AA s in stages 1 and 2 combined (*p* = 0.013). Meanwhile, the excitement/hostility factor scores were significantly different among the three genotypes of rs1011313 in stages 1 and 2 combined (*p* = 0.002, **Table 4**). No significant differences between SNP genotype variations and psychopathology were found at the other 14 SNPs (rs742106, rs16876575, rs4712253, rs9370823, rs9358063, rs3829893, rs2619533, rs4715986, rs12199640, rs2619539, rs2619542, rs9476886, rs1047631, and rs1997679) in stages 1 and 2 combined (*p >* 0.4 for all, **data not shown**).

**Table 4:** Association analyses between SNPs and five factors of PANSS in SZ patients.

SNP	Genotype	N	Total PANSS		Positive		Negative		Depression/anxiety		Cognition		Excitement/hostility	
			Mean	SD	Mean	SD	Mean	SD	Mean	SD	Mean	SD	Mean	SD
rs9464793	AA	487	87.94	19.32	14.29	3.61	23.69	7.68	10.34	5.37	17.51	5.92	15.26	5.52
	AG	70	88.51	19.27	14.87	3.87	23.56	7.91	9.89	6.22	17.69	5.80	15.59	6.48
	GG	4	92.50	6.35	19.25^a^	2.754	30.25	1.50	7.75	5.19	19.25	6.40	14.50	4.20
rs1011313	GG	383	87.22	18.61	14.36	3.76	23.65	7.63	9.80	5.14	17.31	5.96	15.38	5.76
	AG	236	89.47	15.53	14.64	3.25	23.48	6.82	9.67	5.36	18.18	5.29	16.81^b^	5.35
	AA	53	87.77	25.48	13.74	4.23	24.42	8.60	10.51	6.02	18.25	7.00	14.38	5.85

a p = 0.013, compared with AA genotype.

b p = 0.002, compared with each genotype; LSD tests, Bonferroni.

### Genotypes and Medication Efficacy Analysis

We further explored the association between medication efficacy and genotypes. The PANSS scores of 504 SZ patients were tested before and after 6 weeks of antipsychotic medication treatment. The medication efficacy of SZ patients was 374 response and 130 non-response. No association was found between efficacy and genotypes at the 16 SNPs (*p >*0.05 for all, **data not shown**).

### Genotypes and Cognitive Function Analysis

In stage 2, we selected 142 SZ patients and 191 HCs who had finished cognitive function tests, including SDMT, forward-DS, backward-DS, and semantic fluency. We found significant association in the baseline SDMT, forward-DS, backward-DS and semantic fluency between SZ patients and HCs (*p <*0.05 for all, **Supplementary Table 2**). Further, we found association in SDMT and forward-DS between the 6-week and baseline treatments (*p* = 0.002 and 0.0001, respectively). Meanwhile, only SNP rs1011313 genotype had association with the baseline forward-DS (F = 3.41, *p* = 0.036; **data not shown**).

## Discussion

This study aimed to explore the association of *DTNBP1* polymorphisms in patients with SZ and psychopathology and cognition in the Han Chinese population during two stages. We found significant differences in allele frequencies at SNP rs4712253 between SZ patients and HCs. We also found that the rs1011313 genotypes could be associated with excitement/hostility and cognition symptoms in SZ patients.

The present study revealed nominal associations in regard to genotypes and allele frequencies between paranoid SZ patients and HCs at rs742106 in stage 1, and those differences only occurred in male with paranoid SZ. To replicate this finding in other subtypes, we selected an independent sample in stage 2. However, there were no significant associations at rs742106 in stage 2 and stages 1 and 2 combined. These results were consistent with a previous study in Caucasian populations (17). Further, we found a nominal association related to allele frequency between SZ patients and HCs at rs4712253, but previous studies in Caucasian populations showed conflicting results (17). These inconsistent results may be due to population heterogeneity (Caucasian *vs* Chinese population). Also, we found no significant association in relation to genotypes or allele frequency between SZ patients and HCs at rs1011313 and rs2619539; these results were consistent with previous studies and a meta-analysis that included Chinese populations (15, 16, 18–21, 32). Further, although we found significant gender/genotype interaction at SNP rs9370823, and rs2619539 in stages 1 and 2 combined. However, the *P*-value of our results were near 0.05, and these results may be easily reflected type I error. Therefore, further study needs to enlarge the sample size to verify these findings.

Recent meta-analysis revealed that there was no significant association between rs1047631 and SZ in six studies, which in total included 2,764 SZ patients and 3,253 HCs in the European population (33). Our results were consistent with this finding in regard to the Han Chinese population. Meanwhile, previous studies reported that the G allele of rs1047631 may be a risk factor for SZ in the European population (34, 35). However, our results concluded that no association was present in the Han Chinese population. Furthermore, we found no significant association of rs1997679 in SZ patients, this was inconsistent with a previous study (17). However, our results were consistent with a previous study that reported no association of rs9370823, rs3829893, rs1047631, and rs9476886 with SZ (17). Moreover, we found that seven SNPs (rs16876575, rs9464793, rs9358063, rs2619533, rs4715986, rs12199640, and rs2619542) were not associated with SZ that were not reported in the previous study.

There is conflicting available data on th**e** SNPs rs4712253 and rs1047631. These differences may be due to unknown population stratification (15, 17), limited sample size (17), and/or sample heterogeneity (19). To control for these factors, subjects recruited in our study were living in the North Henan provinces and belonged to the same population group based on structure analyses. Moreover, we potentially improved the power to detect disease associations by only selecting paranoid SZ patients in stage 1 and by enlarging the sample size in stage 2. Therefore, we believe our results to be more reliable and consistent than previous studies.

In previous studies, *DTNBP1* haplotypes were found to be risk factors for SZ and protective for male CT-haplotype carriers (14, 15, 36). The study revealed a significant association with SZ with the haplotypes TA and CC, which had an eminent protective effect toward SZ (32). In our study, the haplotypes GAG and GAA in *DTNBP1* were significantly associated with SZ. Although the results were not in line with previous studies, they supported the fact that the haplotypes may be risk factors for SZ.

SZ is characterized by several symptom domains: positive symptoms, negative symptoms, disorganization of thoughts and behaviors, and cognitive deficits (37). Several studies have explored the association between *DTNBP1* and positive symptoms (38), negative symptoms (8), depression symptoms, anxiety symptoms (39), and cognitive deficits (9). In previous studies, analyses of *DTNBP1* tag SNP (e.g., rs1011313) and haplotypes found no conclusive evidence of any association with anxiety or depression disorders (39). Our results were not only consistent with this study, but we also found genotypes of rs1011313 to be associated with excitement and hostility symptoms. A previous study revealed that rs1997679, rs42361617, and rs9370822 were associated with hallucinations, including visual, auditory, and olfactory (38). Therefore, our founding that genotypes of rs9464793 are associated with positive symptoms are also supported.

Cognitive deficits are a core feature of SZ. Meanwhile, a meta-analysis suggested that SZ patients who were minor allele carriers of rs1018381 and rs2619522 had lower cognitive ability scores (9). Similar to a previous study (40), we found that cognitive deficits in SZ patients were associated with genotypes of rs1011313.

This study had several limitations. Our sample size was not large enough to test for cognitive functions. The samples were collected at different times, which meant that the measurement of cognitive function in some samples was not finished. Therefore, further studies are needed with a larger cohort to explore the association between gene variation and cognitive function in SZ. Meanwhile, the statistical power in the current data could be low. Therefore, the improvement of statistical power could better explore the common variation of schizophrenia and further study needs to enlarge the sample size to increase statistical power.

## Conclusion

In summary, this study provides further evidence that SNP rs4712253 of *DTNBP1* has a nominal association with SZ in the Han Chinese population. This genotype variation may play an important role in psychopathology and cognitive function in SZ. Taken together, the results indicate that genetic variations of *DTNBP1* may be associated with SZ and influence psychiatric symptoms.

## Data Availability Statement

The datasets generated for this study are available on request to the corresponding authors.

## Ethics Statement

The studies involving human participants were reviewed and approved by The Ethics Committee of the Second Affiliated Hospital of Xinxiang Medical University. The patients/participants provided their written informed consent to participate in this study.

## Author Contributions

LL designed the study and wrote the protocol. YY, LuZ, LiZ, and WL managed the literature searches and analyses. HY, XS, MSh, MSo, YZ, QL, MD, YL, and GY conducted sample selection and data management. YY, BL, WY, and WL undertook the statistical analysis, and YY, LuZ, DG, XF, and GY wrote the first draft of the manuscript. All authors contributed to and have approved the final manuscript.

## Funding

National Natural Science Foundation of China (81671330, 81971252), the High Scientific and Technological Research Fund of Xinxiang Medical University (2017ZDCG-04), the National Key Research and Development Program of China (2016YFC1307001), the Medical science and technology research project of Henan Province (2018020373), the Science and Technology Project of Henan Province (192102310086), the fund of Henan Clinical Research Center for Mental Disorders, and the support project for the Disciplinary group of Psychology and Neuroscience, Xinxiang Medical University.

## Conflict of Interest

The authors declare that the research was conducted in the absence of any commercial or financial relationships that could be construed as a potential conflict of interest.
