# Supplementary material for: Association of DTNBP1 With Schizophrenia: Findings From Two Independent Samples of Han Chinese Population
Source: Front Psychiatry. 2020 May 25;11:446. doi: 10.3389/fpsyt.2020.00446 (PMC7286384; doi:10.3389/fpsyt.2020.00446)
Supplement: Supplementary file 1 [file Table_1.docx]

**SUPPLEMENTARY TABLE 1** Associated haplotype frequencies of 14 SNPs in the *DTNBP1* gene between SZ and HCs.

| Block | Haplotype | Frequencies | SZ, HCs Frequencies | Chi Square | *P* Value |
| --- | --- | --- | --- | --- | --- |
| 1 | GAG | 0.51 | 0.53, 0.49 | 4.24 | **0.04** |
|  | GAA | 0.25 | 0.23, 0.27 | 6.37 | **0.01** |
|  | AAA | 0.16 | 0.15, 0.16 | 0.09 | 0.76 |
|  | GGG | 0.08 | 0.08, 0.08 | 0.30 | 0.58 |
| 2 | GG | 0.51 | 0.52, 0.49 | 2.71 | 0.09 |
|  | AG | 0.41 | 0.39, 0.42 | 2.62 | 0.10 |
|  | AA | 0.08 | 0.08, 0.08 | 0.02 | 0.88 |
| 3 | GATGGCAG | 0.47 | 0.49, 0.46 | 1.32 | 0.25 |
|  | GATGGCAA | 0.23 | 0.21, 0.24 | 2.02 | 0.15 |
|  | GAAAAGGG | 0.16 | 0.16, 0.17 | 0.55 | 0.45 |
|  | ATAGGGGG | 0.08 | 0.08, 0.07 | 1.54 | 0.21 |
|  | AAAGAGGG | 0.04 | 0.05, 0.04 | 0.63 | 0.42 |

Block 1: rs16876575-rs9464793-rs4712253.

Block 2: rs9370823-rs1997679.

Block 3: rs9358063-rs2619533-rs4715986-rs12199640-rs3829893-rs2619539-rs2619542-rs1011313.
