# Supplementary material for: Association of DTNBP1 With Schizophrenia: Findings From Two Independent Samples of Han Chinese Population
Source: Front Psychiatry. 2020 May 25;11:446. doi: 10.3389/fpsyt.2020.00446 (PMC7286384; doi:10.3389/fpsyt.2020.00446)
Supplement: Supplementary file 2 [file Table_2.doc]

**SUPPLEMENTARY TABLE 2** Associated between genotype and cognition in the *DTNBP1* gene between SZ and HCs.

| Variables | SZ | | p^a^ | HCs | p^b^ |
| --- | --- | --- | --- | --- | --- |
|  | Baseline  (N=142) | Week 6  (N=137) |  | Baseline  (N=191) |  |
| Symbol digital modalities | 33.93±13.43 | 20.75±23.67 | 0.002 | 60.69±12.97 | 0.1×10^-2^ |
| Forward-digital span | 6.65±4.87 | 4.17± 6.79 | 0.1×10^-2^ | 8.95±5.48 | 0.1×10^-2^ |
| Backward-digital span | 4.11± 1.95 | 2.45± 3.01 | 0.081 | 5.88±2.21 | 0.1×10^-2^ |
| Semantic fluency | 13.96± 4.36 | 7.33± 8.27 | 0.092 | 21.38±5.46 | 0.1×10^-2^ |

^a^, *p* value was compare baseline with week 6 in SZ patients.

^b^, *p* value was compare the baseline scores between SZ patients and HCs.
